# The small GTPase Arl8b regulates assembly of the mammalian HOPS complex on lysosomes

**DOI:** 10.1242/jcs.162651

**Published:** 2015-05-01

**Authors:** Divya Khatter, Vivek B. Raina, Devashish Dwivedi, Aastha Sindhwani, Surbhi Bahl, Mahak Sharma

**Affiliations:** Department of Biological Sciences, Indian Institute of Science Education and Research-Mohali (IISERM), India

**Keywords:** Lysosome, Mammalian HOPS complex, hVps41, Arl8b, SKIP

## Abstract

The homotypic fusion and protein sorting (HOPS) complex is a multi-subunit complex conserved from yeast to mammals that regulates late endosome and lysosome fusion. However, little is known about how the HOPS complex is recruited to lysosomes in mammalian cells. Here, we report that the small GTPase Arl8b, but not Rab7 (also known as RAB7A), is essential for membrane localization of the human (h)Vps41 subunit of the HOPS complex. Assembly of the core HOPS subunits to Arl8b- and hVps41-positive lysosomes is guided by their subunit–subunit interactions. RNA interference (RNAi)-mediated depletion of hVps41 resulted in the impaired degradation of EGFR that was rescued upon expression of wild-type but not an Arl8b-binding-defective mutant of hVps41, suggesting that Arl8b-dependent lysosomal localization of hVps41 is required for its endocytic function. Furthermore, we have also identified that the Arl8b effector SKIP (also known as PLEKHM2) interacts with and recruits HOPS subunits to Arl8b and kinesin-positive peripheral lysosomes. Accordingly, RNAi-mediated depletion of SKIP impaired lysosomal trafficking and degradation of EGFR. These findings reveal that Arl8b regulates the association of the human HOPS complex with lysosomal membranes, which is crucial for the function of this tethering complex in endocytic degradation.

## INTRODUCTION

The endocytic system in eukaryotic cells is a complex network of membrane-bound compartments and organelles that constantly exchange material through vesicular or tubular carriers. The Rab and Arf families of small GTPases are important regulators of endocytic trafficking that recruit their numerous effectors to intracellular membranes in a GTP-dependent manner to mediate vesicle budding, tethering and subsequent fusion (Grosshans et al., 2006). Recent studies have also characterized the Arf-like (Arl) family of small GTPases, which are implicated in diverse cellular processes including vesicular trafficking, cytoskeletal organization and ciliogenesis (Burd et al., 2004; Donaldson and Jackson, 2011).

One of the best-understood functions of Rab GTPases is their role in vesicle tethering (Huotari and Helenius, 2011). Rab proteins, in their GTP-bound form, facilitate membrane recruitment of tethering factors that are either long coiled-coil proteins or multi-subunit protein complexes (Kümmel and Heinemann, 2008; Bröcker et al., 2010). For instance, the vacuolar protein sorting pathway in yeast depends upon the multi-subunit tethering complexes (MTCs) class C core vacuole endosome tethering (CORVET) and homotypic fusion and protein sorting (HOPS), which are effectors of the Rab GTPases Vps21 [a Rab5 (also known as RAB5A) homolog] and Ypt7 [a Rab7 (also known as RAB7A) homolog], respectively. Moreover, Rab binding regulates their association with target membranes (Seals et al., 2000; Wurmser et al., 2000; Peplowska et al., 2007; Ostrowicz et al., 2010). Both HOPS and CORVET are hexameric complexes that share four of the six subunits known as the ‘core’ subunits. The core subunits shared by the two MTCs are encoded by the class C phenotypic class of vacuolar protein sorting (VPS) genes and include Vps11p (also known as Pep5), Vps16p, Vps18p and the Sec1-like protein Vps33p. The two ‘accessory’ subunits of HOPS are Vps39 (also known as Vam6) and Vps41 (also known as Vam2), and their corresponding homologous counterparts in CORVET are Vps3 and Vps8 (Wada et al., 1992; Nakamura et al., 1997; Nickerson et al., 2009).

The role of the yeast HOPS complex as a tethering factor required for vacuolar fusion has been extensively studied using both *in vivo* knockout approaches and *in vitro* proteoliposome assays (Hickey and Wickner, 2010; Ostrowicz et al., 2010). Vacuolar localization of the yeast HOPS is mediated by the small GTPase Ypt7 that directly binds to, and recruits Vps41 and Vps39 subunits to vacuolar membranes (Ostrowicz et al., 2010; Bröcker et al., 2012). Once HOPS is targeted to membranes, it catalyzes membrane fusion by recruiting and ‘proofreading’ SNAREs at the fusion site through its Vps33 subunit (Wickner, 2010). Homologs of all six HOPS subunits have also been identified in higher eukaryotes, and studies indicate an evolutionarily conserved function of the metazoan HOPS complex in regulating fusion of endocytic, phagocytic and autophagic cargo with lysosomes and biogenesis of lysosome-related organelles (Sevrioukov et al., 1999; Richardson et al., 2004; Maldonado et al., 2006; Akbar et al., 2011; Swetha et al., 2011; Zlatic et al., 2011; Delahaye et al., 2014; Manil-Ségalen et al., 2014; Takáts et al., 2014).

More recently, several reports have elucidated the role of mammalian HOPS subunits in regulating trafficking towards lysosomes. In summary, human HOPS subunits human (h)Vps41 and hVps39 localize to late endosomes and lysosomes, and their depletion results in accumulation of late endosomes, depletion of lysosomes and a block in degradation of endocytosed cargo (Aoyama et al., 2012; Pols et al., 2013). In agreement with this, previous studies have reported increased endo-lysosome fusion upon overexpression of HOPS subunits (Caplan et al., 2001; Poupon et al., 2003; Pols et al., 2013). Recent studies have shown that, similar to its other homologs, mammalian HOPS subunits not only regulate endocytic traffic but also phagocytic and autophagic traffic towards lysosomes (Barry et al., 2012; Jiang et al., 2014).

Although we now appreciate the importance of the mammalian HOPS complex in regulating cargo delivery to lysosomes, we still do not understand what factors regulate the recruitment of HOPS subunits to lysosomal membranes, and how the HOPS complex assembles on these membranes. Previous reports have suggested that similar to its yeast counterpart, mammalian HOPS subunits interact with Rab7 (also known as RAB7A) and that this interaction regulates recruitment of the HOPS complex to late endosomes and lysosomes. In support of this model, co-immunoprecipitation approaches have shown an association of Rab7 with Vps39 and Vps41 subunits of the mammalian HOPS complex (Rink et al., 2005; Poteryaev et al., 2010; Sun et al., 2010). However, thus far, direct binding to Rab7, and whether any mammalian HOPS subunit acts as a Rab7 effector, has not been demonstrated. Recently, it was also reported that the Rab7 effector RILP (Rab-interacting lysosomal protein) directly interacts with multiple subunits of the HOPS complex and guides their localization to late endosomes (van der Kant et al., 2013; Lin et al., 2014).

Previously, we have found that the hVps41 subunit of the HOPS complex directly interacts with a lysosomal small GTPase of the Arl family, Arl8b, and depletion of Arl8b prevents membrane localization of hVps41 (Garg et al., 2011). Here, we report that the small GTPase Arl8b, but not Rab7 or its effector RILP, targets the HOPS subunit hVps41 to lysosomes. Moreover, depletion of Arl8b dramatically reduced the membrane association of multiple HOPS subunits, suggesting that Arl8b is a crucial factor required for membrane association of the human HOPS complex. In addition, using small interfering RNA (siRNA)-mediated depletion of hVps41 and rescue either with wild-type hVps41 or an Arl8b-binding-defective mutant, we demonstrate that association of hVps41 with Arl8b is required for its function in degradation of endocytic cargo. Previous studies have reported a role for Arl8b and its effector SKIP (also known as PLEKHM2) in regulating kinesin-dependent plus-end-directed motility of lysosomes (Rosa-Ferreira and Munro, 2011). Here, we have identified that the Arl8b effector SKIP directly binds to, and promotes association of Vps39 with Arl8b and kinesin-1-positive peripheral lysosomes, and possibly competes with RILP for association with the HOPS complex. These results suggest that small GTPase Arl8b is a crucial factor that orchestrates the lysosomal assembly of the human HOPS complex and regulates the function of this tethering complex in membrane trafficking.

## RESULTS

### Arl8b, but not Rab7, interacts with the hVps41 subunit of the HOPS complex in a GTP-dependent manner

In our previous study, we identified hVps41 as a direct binding partner for Arl8b, which is recruited to lysosomes in an Arl8b-dependent manner (Garg et al., 2011). However, several reports have implicated a role for Rab7 and/or its effector RILP in regulating late endosomal or lysosomal recruitment of the HOPS complex through interaction with the Vps41 subunit (Sun et al., 2010; van der Kant et al., 2013; Lin et al., 2014).

**Fig. 1.**
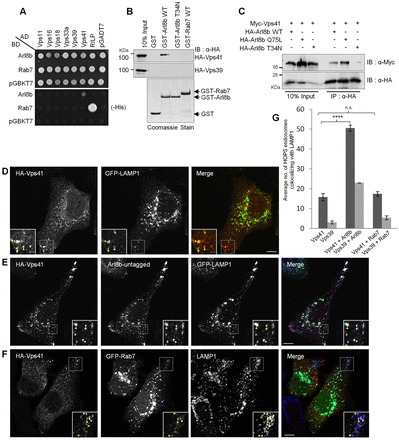
**Arl8b, but not Rab7, interacts with hVps41 and recruits it to lysosomes.** (A) Interaction of the human HOPS subunits with Arl8b and Rab7 was tested in a yeast two-hybrid system by assaying for growth of co-transformants on non-selective medium, to confirm viability, and on selective medium, to detect interactions. BD, binding domain; AD, activating domain. (B) A representative immunoblot (IB) of a GST pulldown assay using HeLa cell lysates expressing either HA–hVps41 or HA–hVps39 incubated with glutathione-conjugated beads bound to the indicated GST proteins. GST-purified proteins were visualized with Coomassie Blue dye staining. WT, wild type. (C) Western blot showing HEK293T cell lysates expressing Myc–hVps41 in combination with different forms of Arl8b, immunoprecipitated (IP) with anti-HA-conjugated beads. (D–G) HeLa cells transfected with HA–hVps41 and GFP–LAMP1 (D), or HA–hVps41, untagged Arl8b and GFP–LAMP1 (E), or HA–Vps41 and GFP–Rab7 (F) were analyzed for lysosomal localization by confocal microscopy, and quantification is shown in G. Colocalized pixels are indicated in the inset. Colocalized puncta are indicated by arrowheads. Values plotted in G correspond to the mean±s.d. of three independent experiments (*n* = 25 cells per experiment); ****P<0.0001; n.s., not significant. Scale bars: 10 µm.

To gain insight into this, we first tested apparent direct interactions of the late endosomal-lysosomal small GTPases Arl8b and Rab7 with the six subunits of the HOPS complex in a yeast two-hybrid assay (Fig. 1A). As reported previously, interaction of Arl8b with the hVps41 subunit was also observed in this assay (Fig. 1A, lower panel) (Garg et al., 2011). No direct interaction of Arl8b was observed with the other five subunits of the HOPS complex. Interestingly, although a strong interaction of Rab7 with its known interaction partner RILP was observed in this assay, no direct interaction was seen with any subunits of the HOPS complex (Fig. 1A, lower panel). These results were further corroborated using a GST pulldown approach in which GST-tagged wild-type Arl8b or Arl8b-T34N (a constitutively GDP-bound mutant) or Rab7 were used as bait to pull down hemagglutinin (HA)–hVps41 from transfected HeLa cell lysates (Fig. 1B, upper panel). The biochemical pulldown data independently confirmed the conclusions from the yeast two-hybrid assay, where interaction of hVps41 was observed with GST–Arl8b, but not with the putative GDP-locked Arl8b-T34N or GST–Rab7. GST pulldown also revealed that both Arl8b and Rab7 do not directly interact with other HOPS subunits (hVps39, hVps16 and hVps18) (Fig. 1B, middle panel; data not shown). Similar to the GST pulldown assay, co-immunoprecipitation from transfected cell lysates also indicated that Vps41 interacts with the wild-type and putative GTP-locked Arl8b mutant (Arl8b-Q75L), but not with the putative GDP-locked Arl8b mutant (Arl8b-T34N, Fig. 1C).

Next, to understand the role of Arl8b versus Rab7 in regulating lysosomal association of hVps41, we overexpressed hVps41 with either of the two GTPases and quantified the number of Vps41 endosomes colocalizing with the late endosome and lysosomal marker LAMP1. HA-tagged hVps41 localizes both to the cytoplasm and peripheral LAMP1-positive vesicles in HeLa cells, as has been reported previously (Fig. 1D). Interestingly, there was a significant increase in the number of Vps41 endosomes colocalizing with LAMP1 in Arl8b-overexpressing cells (Fig. 1E; quantification shown in Fig. 1G). By contrast, no significant change in Vps41 localization was observed upon Rab7 overexpression, with few Vps41-positive structures present on the Rab7- and LAMP1-positive compartment (Fig. 1F; quantification shown in Fig. 1G). hVps39, another lysosomal subunit of the HOPS complex (Caplan et al., 2001; Pols et al., 2013), also showed increased recruitment to LAMP1 endosomes upon overexpression of Arl8b but not Rab7 (quantification shown in Fig. 1G). We also noticed that lysosomes (LAMP2-positive) in Vps41 and Arl8b co-transfected cells appeared to be more clustered and larger than in the untransfected cells, whereas no obvious difference was observed in the distribution of early endosomes (EEA1 positive, supplementary material Fig. S1A,B).

Next, to test whether the GTP-bound form of Arl8b is responsible for hVps41 lysosomal localization, we co-expressed putative GTP-locked (Q75L) and putative GDP-locked (T34N) mutants of Arl8b with hVps41. As predicted, Arl8b-T34N was present in the cytosol and failed to associate with the LAMP1 compartment (merge image, supplementary material Fig. S1D). Unlike the cells transfected with the Arl8b-Q75L mutant, where Vps41 was present on punctate structures, a complete redistribution of Vps41 to the cytoplasm was observed in cells co-expressing the Arl8b-T34N mutant, suggesting that GTP-bound Arl8b is required for membrane localization of hVps41 (merge images, supplementary material Fig. S1C,D).

**Fig. 2.**
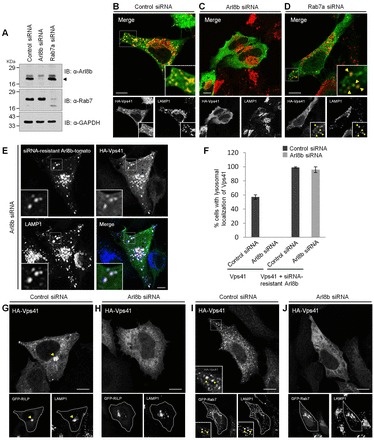
**Arl8b expression is required for hVps41 association with lysosomes and RILP- or Rab7-containing late endosomes.** (A) An immunoblot (IB) of control-, Arl8b- or Rab7-depleted HeLa cell lysates depicting levels of the two proteins. GAPDH was used as the loading control. In the top panel, the arrowhead indicates the position of the dominant band inferred to be Arl8b. (B–E) Immunofluorescence depicting the localization of HA–Vps41 to LAMP1^+^ compartments in control (B), Arl8b- (C) or Rab7- (D) depleted cells, and Arl8b-silenced cells co-expressing HA–Vps41 and siRNA-resistant Arl8b–tomato (E). (F) The percentage of cells with lysosomal localization of Vps41 was quantified following different treatments as indicated. Values plotted are the mean±s.d. of three independent experiments (*n* = 100 cells for each experiment). (G–J) Control (G,I) and Arl8b-siRNA-treated cells (H,J) were co-transfected with HA–Vps41 and either GFP–RILP or GFP–Rab7, stained with LAMP1 and analyzed by confocal microscopy. Colocalized puncta are indicated by arrowheads. Scale bars:10 µm.

Finally, localization of hVps41 was also analyzed in cells treated with control, Arl8b-specific or Rab7-specific siRNA. The efficiency of gene silencing was confirmed by immunoblotting and was found to be >90% for each protein (Fig. 2A). Previous studies have reported that Arl8b silencing prevents kinesin-1-dependent motility of lysosomes to the cell periphery, leading to an accumulation of lysosomes at the microtubule-organizing center (MTOC) (Rosa-Ferreira and Munro, 2011; Tuli et al., 2013). Rab7 silencing causes formation of an enlarged late endosomal compartment with an increased number of intraluminal vesicles (ILVs) as visualized by electron microscopy (Vanlandingham and Ceresa, 2009). Both of these phenotypes were also observed in our experiment upon LAMP1 staining of cells silenced for Arl8b and Rab7 (Fig. 2C,D, red in merge images). Upon analyzing the distribution of HA–hVps41 in the siRNA-treated cells, we found that Vps41 was completely cytosolic in Arl8b-silenced cells (Fig. 2C), but in cells depleted of Rab7, it continued to localize to the LAMP1-positive vesicles (Fig. 2D). Moreover, in Rab7-depleted cells, whereas a known effector of Rab7, RILP, was completely cytosolic (supplementary material Fig. S1E,F), both Vps41 and Arl8b continued to colocalize with the lysosomal marker LAMP1, similar to their localization in control-siRNA-treated cells (supplementary material Fig. S1G,H). Next, to test the specificity of Arl8b depletion, we rescued the effect of Arl8b siRNA by expressing a siRNA-resistant version of Arl8b, and analyzed the localization of Vps41 in the transfected cells. As demonstrated, in siRNA-resistant Arl8b-transfected cells, a complete restoration of Vps41 lysosomal localization was observed (Fig. 2E; quantification is shown in Fig. 2F). We next investigated whether the lack of Vps41 membrane localization observed in Arl8b-siRNA-treated cells could be rescued by co-expressing either Rab7 or RILP, which has previously been shown to directly bind to Vps41 and recruit Vps41 and other HOPS subunits to late endosomes (van der Kant et al., 2013; Lin et al., 2014). In accordance with these studies, we also found strong recruitment of hVps41 to RILP-decorated late endosomes, both in untreated and control-siRNA-treated cells that were clustered near the perinuclear region (Fig. 2G; supplementary material Fig. S1I). Surprisingly, in Arl8b-siRNA-treated cells, although both Rab7 and RILP colocalized with LAMP1 (Fig. 2H,J, lower panels), they failed to recruit Vps41 to these endosomes (Fig. 2H,J, upper panels). This suggests that Arl8b is required for Vps41 lysosomal association and that neither Rab7 nor RILP expression could rescue this effect. Collectively, our findings suggest that Arl8b, but not Rab7 or the Rab7 effector RILP regulates the lysosomal localization of hVps41.

### Interaction of hVps41 with Arl8b requires the N-terminal WD40 domain

**Fig. 3.**
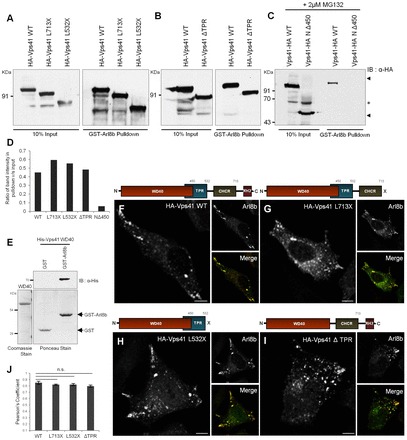
**Interaction of hVps41 with Arl8b requires the N-terminal WD40 domain.** (A–C) A representative immunoblot (IB) of a GST pulldown assay using GST–Arl8b as the bait incubated with HEK293T cell lysates expressing either wild-type (WT) or various domain deletion mutants of Vps41 (as labeled). Treatment with MG132 (2 µM) for 12 hours was performed to restore expression of Vps41-NΔ450. Arrowheads indicate the position of specific bands; the asterisk indicates the position of non-specific bands. (D) Densitometric quantification of two independent GST pulldown experiments with wild-type and mutant Vps41. (E) Purified GST or GST–Arl8b on glutathione–agarose beads were incubated with purified His–Vps41-WD40 domain, subjected to SDS-PAGE and immunoblotted using anti-His. Purified proteins were visualized with Coomassie Blue and Ponceau staining, as indicated. (F–I) HeLa cells were co-transfected with Arl8b and either wild-type HA–Vps41 (F) or HA-tagged domain deletion mutants (G–I, as indicated), and analyzed for their colocalization by confocal microscopy. Scale bars: 10 µm. (J) Pearson's coefficient depicting the overlap of Vps41 with Arl8b was calculated. Values plotted are the mean±s.d. of three independent experiments (*n* = 30 cells per experiment). n.s., not significant.

To gain insight into the mechanisms by which hVps41 interacts with Arl8b, we created several C-terminal domain deletion mutants of hVps41 and analyzed their binding to Arl8b by GST pulldown assays. Bioinformatics analysis predicts three domains within hVps41, namely WD40, clathrin heavy chain repeat (CHCR) and RING-H2 zinc finger domain (see schematic in Fig. 3). A recent study by Harrington et al. has predicted that the amino acids 393–531 of hVps41 form a tetratricopeptide repeat (TPR)-like domain (Harrington et al., 2012). We found that truncation of the RING-H2 zinc finger domain (Vps41-L713X) or truncation of both the RING-H2 and CHCR domains (Vps41-L532X) did not alter the interaction with Arl8b, as observed in the GST pulldown assays (Fig. 3A). Next, we created an internal deletion mutant of Vps41 lacking only the TPR-like domain (Vps41-ΔTPR) and an N-terminal deletion mutant lacking only the WD40 domain (Vps41-NΔ450). Truncation of the complete WD40 domain made the Vps41 protein unstable and possibly a target for proteasomal degradation. Consequently, expression of the Vps41-NΔ450 mutant was only found in cells treated with proteasomal inhibitor, MG132 (Fig. 3C). Interaction analysis using these mutants demonstrated that although deletion of the TPR-like domain did not disrupt binding to Arl8b, Vps41 lacking only the WD40 domain failed to bind to Arl8b (Fig. 3B,C). The data were quantified by densitometric scanning of these blots from two independent experiments to further substantiate these results (Fig. 3D). Furthermore, to confirm that the Vps41 WD40 domain is sufficient for binding to Arl8b, recombinantly expressed, purified His-tagged Vps41 WD40 domain and GST or GST–Arl8b proteins were incubated together in an *in vitro* binding assay. Our results indicate that the Vps41 WD40 domain bound to GST–Arl8b but not GST alone, suggesting that the Vps41 WD40 domain is both essential and sufficient for binding to Arl8b (Fig. 3E).

We next analyzed the localization of the Vps41 truncation mutants in the presence of overexpressed Arl8b to elucidate their recruitment to the Arl8b-positive compartment. As expected from our binding assays, colocalization with Arl8b-positive endosomes was observed with full-length HA-tagged hVps41 and domain deletion mutants lacking RING-H2 (Vps41-L713X) or both RING-H2 and CHCR domains (Vps41-L532X) or only the TPR-like domain (Vps41-ΔTPR) (Fig. 3F–I). Quantification of the colocalization coefficients of Arl8b with wild-type and mutant Vps41 showed no significant differences, indicating that Vps41 association with Arl8b-positive endosomes requires the presence of its WD40 domain (Fig. 3J). The Vps41-NΔ450 mutant was not evaluated in this assay owing to its lack of expression in HeLa cells and rapid degradation by the proteasomal machinery. These results strongly support the importance of the Vps41 WD40 domain in regulating its interaction with Arl8b, and thereby its lysosomal localization and function in endocytic trafficking.

### A T146 SNP in the Vps41 WD40 domain abrogates Arl8b binding and lysosomal localization

**Fig. 4.**
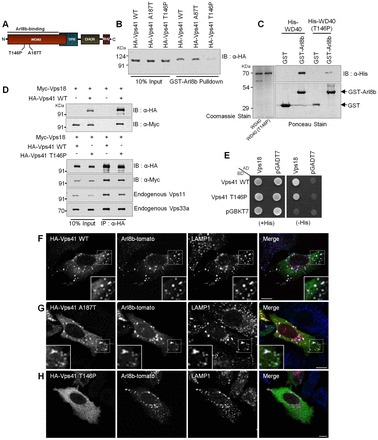
**A T146 SNP in the hVps41 WD40 domain abrogates Arl8b binding and lysosomal localization.** (A) Domain architecture of hVps41, showing the two SNPs in the WD40 domain. (B) A representative immunoblot (IB) of a GST pulldown assay using GST–Arl8b as bait incubated with HeLa cell lysates expressing wild-type (WT) HA–Vps41, HA–Vps41-A187T or HA–Vps41-T146P. (C) Western blot analysis of the purified proteins His–WD40 and His–WD40 (T146P) incubated with either purified GST or GST–Arl8b on beads. (D) Lysates of HEK293T cells transfected with Myc–Vps18 and either HA–Vps41 or HA–Vps41-T146P were immunoprecipitated (IP) using anti-HA antibody followed by anti-Myc immunoblotting. After stripping, the membranes were reprobed with anti-Vps11 and anti-Vps33a antibodies. (E) Yeast two-hybrid interaction of Vps18 was tested with wild-type Vps41 and Vps41-T146P by assaying for growth of co-transformants on non-selective medium, to confirm viability, and on selective medium, to detect interactions. AD, activating domain; BD, binding domain. (F–H) Immunostaining with LAMP1 of HeLa cells co-transfected with Arl8b–tomato and either wild-type HA–Vps41 (F) or HA–Vps41-A187T (G) or HA–Vps41-T146P (H). Colocalized pixels are shown in the inset. Scale bars: 10 µm.

Previous studies have identified hVps41 as a candidate gene mediating neuroprotection of *Caenorhabditis elegans* dopaminergic neurons from α-synuclein-induced neurodegeneration (Ruan et al., 2010). Moreover, two SNPs within the WD40 domain of hVps41 (A187T and T146P) were identified that abrogate the neuroprotective effect of Vps41 (Harrington et al., 2012). Because our results indicate that the WD40 domain of hVps41 is important for Arl8b-binding, we wanted to determine the consequence of these two SNPs within this domain on Arl8b-binding and subsequent recruitment to lysosomes (Fig. 4A). Although there was no difference observed in the interaction of Vps41-A187T with GST–Arl8b as compared to wild-type Vps41, the Vps41-T146P version showed a dramatic reduction in binding to GST–Arl8b (Fig. 4B). Further confirmation of these results was performed using purified proteins where either His-tagged wild-type WD40 or WD40-T146P was incubated with GST and GST–Arl8b (Fig. 4C). Significant loss of binding of WD40-T146P to GST–Arl8b, in comparison to the wild-type WD40 domain, further confirmed that this SNP in Vps41 abrogates its binding to Arl8b. To test whether the impaired binding to Arl8b might be due to the unfolding of the Vps41 led by the T146P conversion, circular dichroism experiments were carried out with purified wild-type and T146P WD40 domains. Circular dichroism spectroscopy data indicated that this substitution led to structural changes in the WD40 domain, but not a complete denaturation of the overall secondary structure of this domain or random coil formation (data not shown). In support of this argument, the T146P substitution in hVps41, despite impairing binding to Arl8b, did not change association with other HOPS subunits as concluded from both co-immunoprecipitation and yeast two-hybrid approaches (Fig. 4D,E). We also expressed these hVps41 SNP transgenes to test their recruitment to Arl8b-positive lysosomes. As expected from loss of binding to Arl8b, Vps41-T146P also failed to be recruited to the Arl8b and LAMP1 compartment and continued to be cytosolic in the presence of overexpressed Arl8b, whereas both wild-type hVps41 and A187T showed strong colocalization with Arl8b (Fig. 4F–H). These observations explain how SNP T146P becomes deleterious to the neuroprotective function of hVps41, as this substitution prevents lysosomal localization of hVps41 in an Arl8b-dependent manner, even though it does not disrupt Vps41 association with other HOPS subunits.

### Subunit–subunit interactions guide assembly of human HOPS subunits on Arl8b-positive lysosomes

Given the role of Arl8b in regulating the lysosomal association of hVps41, we next investigated how other subunits of the human HOPS complex are recruited to Arl8b- and hVps41-positive lysosomes. We hypothesized that the recruitment of other HOPS subunits must be guided by their topological arrangement within the complex. To address this question, we first tested the interaction of human HOPS subunits with each other in a yeast two-hybrid assay (supplementary material Fig. S2A). Our results suggest a model of human HOPS complex arrangement that is strikingly similar to that of its yeast counterpart (Bröcker et al., 2012) (supplementary material Fig. S2B). In particular, the interactions of hVps41 with hVps18, of hVps39 with hVps11, and of hVps16 with hVps33a were also conserved in mammalian cells (supplementary material Fig. S2A).

**Fig. 5.**
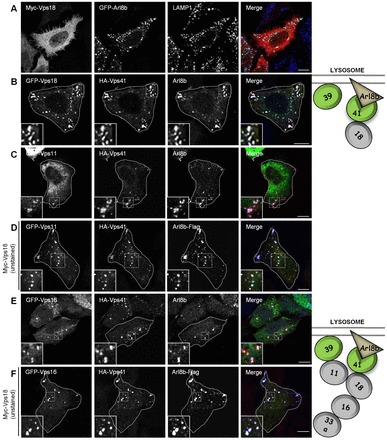
**Subunit–subunit interactions guide assembly of the human HOPS complex to Arl8b-positive lysosomes.** (A–F) Confocal micrographs depicting the localization of the indicated HOPS subunits to Arl8b^+^ and LAMP1^+^ compartments upon overexpression in multiple combinations in HeLa cells. Colocalized pixels are shown in the inset. Scale bars: 10 µm.

Next, we tested whether the assembly of other HOPS subunits on Arl8b- and hVps41-positive lysosomes is directed by their protein–protein interactions. We first analyzed recruitment of the hVps18 to Arl8b- and hVps41-positive endosomes. Although hVps18 had no significant colocalization with Arl8b when expressed alone, it was completely recruited to Arl8b structures upon co-expression with hVps41, suggesting that hVps41 recruits hVps18 to lysosomal membranes (Fig. 5A,B). Unlike hVps18, hVps11 and hVps16 subunits were not efficiently recruited to Arl8b structures even in the presence of overexpressed hVps41 and continued to be largely cytosolic (Fig. 5C,E). Only a few punctate structures that showed colocalization with Arl8b and hVps41 were present in these cells. Our yeast two-hybrid interaction analysis agrees with these observations, as neither hVps11 nor hVps16 show direct interaction with hVps41, but rather interaction with hVps18 (supplementary material Fig. S2A). Thus, as predicted from our interaction data, co-expression of hVps18 along with hVps41 and Arl8b led to dramatic recruitment of both hVps11 and hVps16 to Arl8b-positive lysosomes (Fig. 5D,F, see quantification in supplementary material Fig. S2C). Taken together, these results support our hypothesis that the assembly of HOPS complex on Arl8b-containing lysosomes in the cell is guided by their subunit–subunit interactions.

### Arl8b is required for membrane localization of human HOPS subunits

**Fig. 6.**
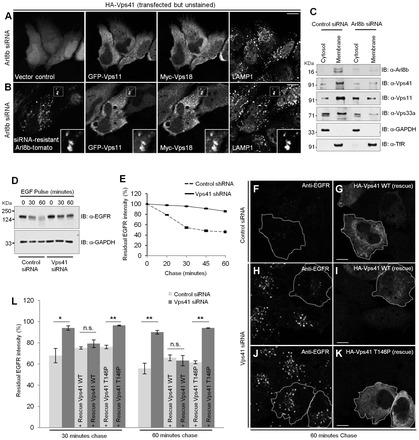
**Arl8b is required for membrane localization of human HOPS subunits, and for Vps41 function in endocytic degradation of EGFR.** (A,B) Arl8b-silenced HeLa cells were transfected with either ptd-Tomato-N1 vector (A) or siRNA-resistant Arl8b–tomato construct (B) together with the HOPS subunits GFP–Vps11, Myc–Vps18 and HA–Vps41 (unstained), and analyzed for their localization to Arl8b^+^ lysosomal compartments. Scale bars: 10 µm. Colocalized pixels are shown in the inset. (C) Immunoblotting (IB) depicting the presence of endogenous HOPS subunits in membrane and cytosol fractions of control- and Arl8b-siRNA-treated HeLa cells. GAPDH and TfR were used as markers for cytosol and membrane fractions, respectively. (D) Serum-starved control or Vps41-silenced HeLa cells were pulsed with unlabeled EGF (100 ng/ml) for the indicated time periods. The expression of EGFR and GAPDH (loading control) were detected by immunoblotting of whole-cell extracts. (E) Serum-starved control or Vps41-silenced HeLa cells were pulsed with Rhodamine–EGF (500 ng/ml) for 7 minutes and chased in complete medium for varying times. EGFR degradation was assessed by measuring residual EGFR intensity over these time periods. Values plotted are the mean±s.d. of three independent experiments (*n* = 60 cells for each time point in every experiment). (F–K) Control (F,G) or Vps41-silenced HeLa cells (H–K) were either transfected with siRNA-resistant wild-type (WT) HA–Vps41 (F–I), or with siRNA-resistant HA–Vps41-T146P (J,K), and EGFR degradation was monitored after 30 and 60 minutes of chase by staining with anti-EGFR antibody. Shown are representative images of EGFR trafficking to lysosomes after 60 minutes of chase. Scale bars: 10 µm. (L) Quantification of the percent residual EGFR fluorescence intensity in either case was performed for each indicated time point. Values plotted are the mean±s.d. of three independent experiments (*n* = 100 cells for each time point per experiment); *P<0.05; **P<0.01; n.s., not significant.

To investigate whether Arl8b is required for localization of the multi-subunit human HOPS complex on lysosomes, we expressed HOPS subunits hVps41, hVps18 and hVps11 in Arl8b-siRNA-treated cells that were either transfected with vector control or with siRNA-resistant Arl8b construct, and analyzed their colocalization with LAMP1. Similar to our results with Vps41, we found that, in Arl8b-depleted cells, multiple HOPS subunits were localized to the cytoplasm (Fig. 6A). Although this cytoplasmic localization of HOPS subunits was not rescued by expression of vector control, it was completely rescued in cells transfected with the siRNA-resistant Arl8b construct, and HOPS subunits colocalized with the LAMP1 compartment in these cells (Fig. 6A,B, see inset). To confirm these observations under endogenous conditions, we performed fractionation to test the membrane association of HOPS subunits in control- versus Arl8b-siRNA-treated cells. As depicted in Fig. 6C, HeLa cells treated with Arl8b siRNA showed a dramatic decrease in membrane-associated hVps41, hVps11 and hVps33a levels as compared to those of control-siRNA-treated cells (Fig. 6C, compare lanes 2 and 4). These results strongly support our conclusion that Arl8b is required for lysosomal recruitment of the HOPS complex.

### Depletion of hVps41 results in delayed trafficking and degradation of EGFR in lysosomes

A recent study has highlighted the roles of hVps41 and hVps39 in regulating cargo traffic to lysosomes by mediating homotypic and heterotypic late endosome fusion (Pols et al., 2013). Our results from this study suggest that Arl8b regulates lysosomal localization of the HOPS complex, implying that Arl8b is an important regulator of HOPS function in endocytic traffic. To address this, we first monitored the endocytic degradation of EGFR as a model to study the role of hVps41 in this pathway. HeLa cells were treated with control siRNA or hVps41 siRNA or transduced with lentivirus particles containing scrambled short hairpin RNA (shRNA) (control) or shRNA against hVps41. The efficiency of hVps41 depletion using either siRNA or shRNA treatment was found to be >85% as determined by western blotting (supplementary material Fig. S2D,E). We monitored the endocytic degradation of EGFR in hVps41-depleted cells by stimulating the cells with EGF and analyzing levels of EGFR in total cell lysates at different time points. In hVps41-siRNA-treated cells, EGFR degradation was significantly delayed compared to that of control-siRNA-treated cells (Fig. 6D). We also monitored the levels of EGFR remaining by using immunofluorescence after incubating control- and hVps41-shRNA-transduced cells with EGF followed by chase for various time periods in complete medium. Whereas in control-shRNA-transduced cells, EGFR signal was significantly reduced by 60 minutes of chase, most of the EGFR signal in hVps41-shRNA-transduced cells persisted during these time points, indicating that EGFR degradation is delayed in hVps41-depleted cells (merge images, supplementary material Fig. S2F–K). Quantification of these images showed an approximately twofold EGFR signal remaining in hVps41-depleted cells compared to that of control cells at 60 minutes of chase (Fig. 6E). Furthermore, colocalization of internalized EGF with the early endosomal marker EEA1 and with endocytosed Alexa-Fluor-488-labeled dextran (to label lysosomes) was also assessed in control versus Vps41-depleted cells. In comparison to control cells, in Vps41-depleted cells, EGF failed to reach the dextran compartment by 30 minutes of chase and several EGF-containing endosomes remained positive for EEA1, indicating that there is delay in EGFR trafficking to lysosomes upon depletion of Vps41 (supplementary material Fig. S3A–D, see the arrowheads; quantification of colocalization between EGF and dextran is shown in supplementary material Fig. S3E).

### Interaction of hVps41 with Arl8b is required for rescue of endocytic degradation of EGFR in hVps41-depleted cells

To elucidate whether interaction of Vps41 with Arl8b is crucial for its function in endocytic trafficking, we complemented control and hVps41-depleted cells with siRNA-resistant cDNAs for wild-type hVps41 or an Arl8b-binding-defective mutant form (hVps41-T146P) and monitored the EGFR signal remaining by pulse-chase experiment. Whereas wild-type hVps41 was able to efficiently rescue the delay in EGFR degradation, the hVps41-T146P mutant, which does not interact with Arl8b and therefore is not recruited to lysosomal membranes, failed to rescue the effect of hVps41 depletion on EGFR degradation (Fig. 6F–K; quantification of images shown in Fig. 6L). These results clearly demonstrate that interaction of hVps41 with Arl8b is crucial for function of hVps41 and probably of the human HOPS complex in the endocytic degradation pathway.

### The Arl8b effector SKIP directly interacts with the Vps39 subunit of the HOPS complex and recruits it to Arl8b-positive lysosomes

Although our finding that Arl8b recruits the HOPS complex to lysosomes explains the reported function of Arl8b in regulating cargo traffic to lysosomes, another profound and probably better-understood function of Arl8b is in controlling microtubule-based lysosomal motility (Hofmann and Munro, 2006). Arl8b in its GTP-bound form interacts with SKIP/PLEKHM2, which directly binds to kinesin light chain (KLC2) and recruits kinesin to drive plus-end-directed movement of lysosomes towards the cell periphery (Rosa-Ferreira and Munro, 2011). Thus, the Arl8b–SKIP–KLC2 complex functions in an opposing manner to the Rab7–RILP–p150^Glued^ complex, which promotes minus-end-directed transport of lysosomes. Interestingly, recent studies have shown that the Rab7–RILP complex associates with multiple HOPS subunits and this interaction is thought to couple minus-end transport and tethering steps during late endocytic trafficking (van der Kant et al., 2013). However, whether a similar cross-talk exists for the transport and vesicle tethering functions regulated by Arl8b, and how this affects the association of the HOPS complex with RILP is not understood.

**Fig. 7.**
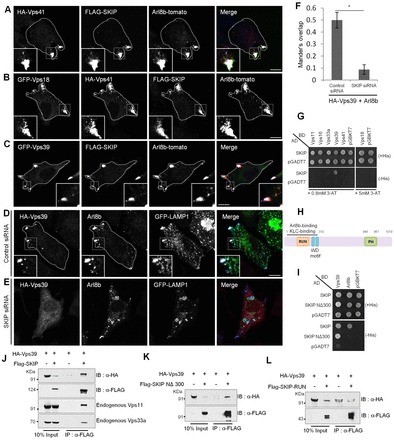
**SKIP directly interacts with the Vps39 subunit of the HOPS complex, and recruits it to Arl8b-positive lysosomes.** (A–C) Representative confocal micrographs from HeLa cells transfected with the indicated constructs. The boundary of the transfected cell is highlighted to visualize the peripheral position of the colocalized endosomes. (D–F) Immunofluorescence of control or SKIP-silenced HeLa cells transfected with HA–Vps39 and Arl8b. Scale bars: 10 µm. Colocalization was analyzed using Mander's method over three independent experiments (*n* = 30 cells per experiment); data show the mean±s.d.; *P<0.05. (G) Interaction of human HOPS subunits with SKIP was tested in a yeast two-hybrid system by assaying for growth of co-transformants on non-selective medium, to confirm viability, and on selective medium, to detect interactions. AD, activating domain; BD, binding domain. (H) Domain architecture of SKIP. (I) Yeast two-hybrid interaction of Vps39 and Arl8b was tested with SKIP and SKIP ΔRUN by assaying for growth of co-transformants on non-selective medium to confirm viability and on selective medium to detect interactions. (J–L) Lysates from HEK293T cells co-transfected with HA–Vps39 and Flag–SKIP wild-type or truncation mutants (as indicated), were subjected to immunoprecipitation (IP) with anti-Flag antibodies, and immunoblotted (IB) with HA antibodies to detect the interactions. After stripping, the membranes were re-probed with anti-Vps11 and anti-Vps33a antibodies (J).

We found strong colocalization of Arl8b–Vps41 and Arl8b–Vps41–Vps18 with SKIP on peripheral structures (Fig. 7A,B). A similar colocalization of Arl8b and SKIP was also observed with the hVps39 subunit of the HOPS complex with all three proteins present on the same compartment (Fig. 7C). Furthermore, KLC2 was also entirely recruited to the HOPS–Arl8b–SKIP-positive compartment, which explains their spatial location towards the cell periphery (supplementary material Fig. S4A,B). Arl8b expression was found to be crucial for the association of HOPS subunits with SKIP-positive structures, as siRNA against Arl8b led to a cytosolic redistribution of both Vps41 and Vps39, and their colocalization with SKIP was greatly reduced (supplementary material Fig. S4C–F). Not surprisingly, SKIP also appeared to be more cytosolic in Arl8b-siRNA-treated cells (supplementary material Fig. S4D,F), as Arl8b has been previously implicated in the recruitment of SKIP to lysosomes (Rosa-Ferreira and Munro, 2011).

Next, to investigate the role of SKIP in regulating the HOPS complex, we treated cells with either control or SKIP siRNA and scored their effect on the colocalization of Arl8b and HOPS subunits Vps41 and Vps39. The efficiency of SKIP depletion was found to be >85% as measured by qRT-PCR (supplementary material Fig. S4G). Interestingly, although no change was observed in the colocalization of Vps41 and Arl8b upon SKIP depletion (data not shown), recruitment of Vps39 to the Arl8b- and LAMP1-positive compartment was strongly reduced in the SKIP-siRNA-treated cells compared to that of controls, indicating that SKIP is a crucial linker that regulates Vps39 recruitment to Arl8b-positive lysosomes (Fig. 7D,E; see quantification graph, Fig. 7F).

This led us to test whether there is a direct binding of SKIP with the hVps39 subunit of the HOPS complex, which might lead to recruitment of Vps39 to Arl8b-positive lysosomes. Therefore, we tested the interaction of SKIP with the six subunits of the HOPS complex in a yeast two-hybrid assay. Interestingly, strong binding of SKIP with only the hVps39, but not other HOPS subunits, was observed (Fig. 7G). Previous studies have reported that interaction of Arl8b and KLC2 with SKIP requires the RUN domain and W-acidic motifs, respectively (Rosa-Ferreira and Munro, 2011; Pernigo et al., 2013). Our results here indicate that the binding of SKIP to Vps39 does not require its N-terminal RUN domain or W-acidic motifs as this interaction was also found with a domain deletion mutant of SKIP, NΔ300, lacking these regions. As reported previously, Arl8b interaction was observed with full-length SKIP, but not with SKIP-NΔ300 (Fig. 7H,I). These results were further corroborated by co-immunoprecipitation experiments, which clearly indicated a strong interaction of Vps39 with SKIP *in vivo* that is not dependent upon the RUN domain or W-acidic motifs of SKIP (Fig. 7J–L). Notably, we also found that endogenous HOPS subunits Vps11 and Vps33a were also present in this complex of SKIP and Vps39, indicating that SKIP interacts with the HOPS complex through Vps39 (Fig. 7J).

**Fig. 8.**
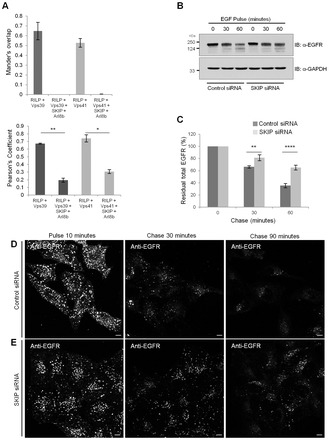
**SKIP competes with RILP for association with HOPS subunits, and depletion of SKIP results in delayed EGFR degradation in lysosomes.** (A) Colocalization of HA–Vps39 and HA–Vps41 with GFP–RILP in transfected HeLa cells was assessed by measuring the Mander's and Pearson's coefficients. Values plotted correspond to the mean±s.d. of three independent experiments (*n* = 30 cells per experiment); *P<0.05; **P<0.01. (B) Control or SKIP-silenced HeLa cells were serum starved and pulsed with unlabeled EGF for the indicated time periods. The expression of EGFR and GAPDH (loading control) was detected by immunoblotting (IB) of whole-cell extracts. (C) Densitometric analysis of the residual total EGFR signal relative to GAPDH was performed for five independent experiments. Plotted values correspond to the mean±s.d. **P<0.01; ****P<0.0001. (D,E) Control (D) or SKIP-silenced (E) HeLa cells were serum starved, pulse-chased with unlabeled EGF for the indicated time periods, fixed for immunofluorescence and labeled with anti-EGFR. Scale bars: 10 µm.

The Rab7 effector RILP interacts with multiple HOPS subunits, including Vps41 and Vps39, and recruits them to late endosomes. RILP- and HOPS-complex-positive late endosomes cluster at the MTOC, as direct interaction of RILP with the dynactin–dynein complex drives these endosomes towards the minus end of microtubules (van der Kant et al., 2013) (also see supplementary material Fig. S4H,I). To understand how the presence of SKIP regulates the association of HOPS with RILP, we compared colocalization between RILP and hVps41 or Vps39 in cells with or without SKIP overexpression. A complete lack of colocalization between RILP and either Vps41 or Vps39 was observed in the presence of overexpressed Arl8b and SKIP, and HOPS subunits accumulated at the cell periphery with Arl8b and SKIP, whereas RILP retained its perinuclear distribution (see quantification of colocalization coefficients in Fig. 8A and supplementary material Fig. S4J,K). These results suggest that the Arl8b–SKIP complex competes with the Rab7–RILP complex for interaction with HOPS subunits and that the two GTPases Arl8b and Rab7 likely regulate motility of the HOPS-positive late endosomes and lysosomes towards opposite ends of microtubules.

### SKIP/PLEKHM2 depletion results in delayed trafficking and degradation of EGFR in lysosomes

Our results shown here indicate that the Arl8b effector SKIP plays a direct role in recruiting HOPS subunits to lysosomes. As the association of Arl8b with HOPS is crucial for endocytic degradation of EGFR, we tested whether SKIP also regulates EGFR trafficking to lysosomes. We monitored EGFR turnover in SKIP-depleted cells as explained for Fig. 6D. Indeed, in SKIP-siRNA-treated cells, EGFR degradation was significantly delayed at 60 minutes compared to that of control-siRNA-treated cells (Fig. 8B,C). A similar delay in EGFR degradation upon SKIP depletion was also observed by immunofluorescence when cells were pulsed with unlabeled EGF followed by chase for 30 and 90 minutes in complete medium (Fig. 8D,E). These results indicate that, similar to the HOPS complex, SKIP also positively regulates the trafficking and lysosomal degradation of EGFR. In conclusion, our results show that Arl8b orchestrates the membrane association and endocytic function of HOPS subunits by directly recruiting Vps41 to lysosomes and, indirectly, by association of its effector SKIP with Vps39.

## DISCUSSION

The HOPS complex is a highly conserved multi-subunit tethering factor that regulates fusion of late endosomes and lysosomes, and thereby cargo degradation in lysosomes. Despite several studies supporting the crucial role of mammalian HOPS subunits in regulating the endocytic degradation pathway, little is known about how this complex is recruited to lysosomes and how its activity is regulated.

In *Saccharomyces cerevisiae* (where there is no Arl8b homolog), the Rab7 homolog Ypt7 interacts with Vps41p and Vps39p and recruits the HOPS complex to vacuolar membranes (Hickey et al., 2009). Previous studies had hypothesized that, similar to yeast, Rab7 also regulates HOPS complex membrane localization in higher eukaryotes. In this study, we have demonstrated that the small GTPase Arl8b mediates recruitment of the human HOPS complex to lysosomal membranes. We noted that Arl8b, but not Rab7, directly interacts with the hVps41 subunit of the HOPS complex and regulates its association with lysosomes (Fig. 1). We also found that GTP-bound Arl8b was essential for lysosomal localization of hVps41, and that neither Rab7 nor its effector RILP (which was previously shown to recruit Vps41 to late endosomes) was able to rescue hVps41 membrane localization in Arl8b-depleted cells (Fig. 2; supplementary material Fig. S1). Our results are further corroborated by a recent study showing evidence of a physical interaction of Arl8b and Vps41 in *C. elegans* and their coordinated function in phago-lysosome formation (Sasaki et al., 2013).

In a previous study by Harrington et al., two SNPs in the WD40 domain of hVps41 were reported to cause a loss-of-function phenotype in neuroprotection from α-synuclein-induced neurodegeneration in *C. elegans* and neuroglioma cells (Harrington et al., 2012). Our results indicate that one of these substitutions, T146P, causes a dramatic loss of binding to Arl8b and prevents hVps41 localization to lysosomes (Fig. 4). Moreover, this SNP in hVps41 also led to impairment in hVps41 function during endocytic degradation of EGFR (Fig. 6). Collectively, our findings indicate that Arl8b-dependent localization of hVps41 to lysosomes is crucial for its function in endocytic trafficking.

Thus far, no studies have reported how the mammalian HOPS complex assembles on lysosomal membranes and what regulates membrane recruitment of this complex. Our results indicate that direct interaction of hVps41 with hVps18 and, similarly, of hVps18 with hVps11 and hVps16, regulate their recruitment to Arl8b- and LAMP1-positive endosomes (Fig. 5; supplementary material Fig. S2). Moreover, using knockdown approaches, we demonstrated that Arl8b is essential for membrane localization of the multiple subunits of the human HOPS complex (Fig. 6). These results support a model whereby human HOPS subunits undergo hierarchical assembly on Arl8b-positive lysosomes guided by their subunit–subunit interactions.

The small GTPase Arl8b has been shown previously to mediate recruitment of the molecular motor kinesin-1 through its effector SKIP on lysosomal membranes (Rosa-Ferreira and Munro, 2011). This tripartite complex of Arl8b–SKIP–KLC2 is implicated in plus-end movement of lysosomes towards the cell periphery. Interestingly, our results presented here indicate that HOPS subunits also associate with this Arl8b–SKIP–KLC2 complex and localize to the cell periphery. Moreover, we found a novel interaction of SKIP with the HOPS subunit Vps39 that regulates the recruitment of Vps39 to Arl8b- and LAMP1-positive endosomes (Fig. 7; supplementary material Fig. S4).

Given that multi-subunit tethering factors are proposed to bridge the two membranes prior to vesicle fusion and are predicted to have two membrane-binding sites, we propose a model whereby interaction of Arl8b on lysosomal membranes and of Rab7–RILP on late endosomes with the HOPS complex brings the two compartments into close proximity for fusion. Moreover, recruitment of the molecular motors dynein and kinesin-1 by Rab7 and Arl8b effectors, respectively, to HOPS-containing endosomes drives motility of the HOPS-positive late endosomes and lysosomes in opposite directions. In agreement with this, we found that co-expression of SKIP and Arl8b prevents localization of HOPS subunits to the RILP-positive compartment, suggesting a competition between these two endocytic machineries.

In summary, our data clearly implicates the lysosomal small GTPase Arl8b in regulating the recruitment of human HOPS subunits to lysosomes through its direct interaction with hVps41 and through interaction of its effector SKIP with the hVps39 subunit of the HOPS complex. Assembly of the core HOPS subunits on lysosomes is guided by their subunit–subunit interactions. These findings implicate the human HOPS complex as an Arl8b effector, and implicate Arl8b as a crucial regulator of function of the HOPS complex in membrane trafficking. Further elucidation of the Arl8b–HOPS interaction in the context of phagocytic and autophagic pathways might shed light on how the HOPS complex is recruited to these intracellular membranes.

## MATERIALS AND METHODS

### Plasmids

Vps expression constructs were kind gifts from Drs Chengyu Liang (University of Southern California, Los Angeles, USA), J. Wade Harper (Harvard Medical School, Boston, USA) and Victor Faundez (Emory University, Atlanta, USA). GFP–Rab7, GFP–Rab7-T22N and GFP–LAMP1 expression constructs were kind gifts from Dr Steve Caplan (University of Nebraska Medical Center, Omaha, USA). The GFP–RILP plasmid was a kind gift from Dr Jacques Neefjes (The Netherlands Cancer Institute, Amsterdam, Netherlands). GFP–KLC2 plasmid was a kind gift from Dr Michael Way (London Research Institute, London, UK). The following constructs were described previously: wild-type Arl8b, Q75L and T34N in pcDNA3.1, GFP-Arl8b, Arl8b-pGEX-4T3, Arl8b-T34N-pGEX-4T3, Rab7-pGEX-4T3, Arl8b-pGBKT7 and Vps41-pGADT7 (Garg et al., 2011). Arl8b with C-terminal HA, Myc or Flag epitopes, Flag-SKIP-WT, NΔ300, Flag-SKIP-RUN (1–300) and HA-Vps41-NΔ450 were cloned in pcDNA3.1(-) (Invitrogen). Arl8b-tomato expression constructs were cloned in ptd-Tomato-N1 (Clontech) vector. The human HOPS subunits and SKIP yeast two-hybrid plasmids were cloned in pGBKT7 and pGADT7 vectors (Clontech). All the point mutants, truncation mutants and siRNA-resistant constructs were constructed using Stratagene site-directed mutagenesis kit (Agilent). HA-Vps41-ΔTPR was made by overlap extension PCR. For transient transfections, cells grown on glass coverslips were transfected with the desired constructs using X-tremeGENE HP DNA reagent (Roche) for 18–20 hours.

### Reagents and antibodies

The reagents used in this study are as follows: human EGF, Rhodamine-labeled EGF, dextran–Oregon-Green (Life Technologies) and MG132 (Sigma). The following antibodies were used in this study: anti-LAMP1, anti-LAMP2, anti-EEA1 (BD Biosciences), rabbit anti-LAMP1 (Novus Biologicals), anti-Rab7 (Cell Signaling Technology), anti-Vps41, anti-EGFR, anti-GAPDH (Santa Cruz Biotechnology), anti-Vps11 (Abcam), anti-Vps33a (Proteintech), anti-EGFR, anti-TfR, anti-Myc (Life Technologies), anti-β-actin, anti-tubulin, anti-Flag (Sigma), anti-HA (Covance) and anti-His (Pierce). Alexa-Fluor-conjugated secondary goat anti-mouse-IgG and goat anti-rabbit-IgG antibodies were purchased from Life Technologies. Goat anti-mouse-IgG and anti-rabbit-IgG horseradish-peroxidase-conjugated antibodies were obtained from Jackson ImmunoResearch Laboratories.

### Cell culture and microscopy

The HeLa and HEK293T cells were cultured in DMEM (Life Technologies) supplemented with 10% FBS (Life Technologies) in a 5% humidified incubator at 37°C. Cells were immunostained as described previously (Sharma et al., 2008). Single-plane confocal images were acquired with a Zeiss 710 Confocal Laser Scanning Microscope using a Plan-Apochromat 63×/1.4 NA oil objective with appropriate filters. The Zeiss Zen software was used for data acquisition.

### Protein purification and GST pulldown assay

GST, GST–Arl8b, GST–Arl8b-T34N, GST–Rab7 and His–Vps41-WD40 were purified using standard methods, and GST pulldown assays and purified protein–protein interaction assays were performed as described previously (Garg et al., 2011).

### Cell lysates, co-immunoprecipitation, membrane-cytosol fractionation and immunoblotting

For lysates, cells were lysed in ice-cold lysis buffer (25 mM Tris-HCl pH 7.4, 150 mM NaCl, 1 mM EDTA, 1% Triton X-100 and protease inhibitor cocktail) or ice-cold RIPA buffer for the EGFR degradation assay. For co-immunoprecipitation, transfected HEK293T cells were lysed in ice-cold lysis buffer (20 mM Tris-HCl pH 7.4, 150 mM NaCl, 0.5% NP-40, 1 mM sodium orthovandate). The membrane-cytosol fractionation was performed as described previously (Sharma et al., 2008). Immunoblotting was performed as described previously (Garg et al., 2011).

### Yeast two-hybrid analysis

The yeast two-hybrid assay was performed as described previously (Sharma et al., 2009). Briefly, the *S. cerevisiae* strain AH109 co-transformants were streaked on double-dropout plates lacking leucine and tryptophan, allowed to grow at 30°C for 3 days and replated on non-selection (+HIS) and selection plates (−HIS).

### Gene silencing by siRNA and shRNA

siRNA duplexes for non-targeting siRNA, Arl8b (5′-AGGTAACGTCACAATAAAGAT-3′), Vps41 (5′-CCATTGACAAACCACCATTTA-3′), Rab7 and SKIP (ON-TARGETplus SMARTpool) were purchased from Dharmacon, and the knockdown was performed as per the manufacturer's instructions. For shRNA-mediated gene silencing, knockdown was performed as described previously (Tuli et al., 2013). shRNA target sequences were as follows: Mission shRNA, 5′-CAACAAGATGAAGAGCACCAA-3′; Vps41 shRNA, 5′-CCATTGACAAACCACCATTTA-3′.

### EGFR degradation assay

Cells were incubated with EGF at 37°C and chased in complete medium at 37°C for the indicated time periods, after which they were processed for either immunofluorescence or western blotting (as described above). For quantification of EGFR intensity, images were imported into ImageJ software (NIH). Total cell fluorescence (CTCF) of remaining EGFR was calculated using the formula CTCF = integrated density−(area×mean fluorescence of background).

### Statistics

For calculation of Pearson's and Mander's coefficients, images were imported into ImageJ and fluorescence levels were set to a threshold to optimally depict punctate structures. Densitometry was performed using the Adobe Photoshop software. Statistical significance was calculated using paired Student's *t*-tests.

## Supplementary Material

Supplementary Material
