# Supplementary material for: The small GTPase Arl8b regulates assembly of the mammalian HOPS complex on lysosomes
Source: J Cell Sci. 2015 May 1;128(9):1746–61. doi: 10.1242/jcs.162651 (PMC4432227; doi:10.1242/jcs.162651)
Supplement: Supplementary Material [file supp_128_9_1746__index.html]

The small GTPase Arl8b regulates assembly of the mammalian HOPS complex on lysosomes — Supplementary Material 

# The small GTPase Arl8b regulates assembly of the mammalian HOPS complex on lysosomes

## JCS162651 Supplementary Material

**Files in this Data Supplement:**

- **Supplementary Material**
