# Supplementary material for: The small GTPase Arl8b regulates assembly of the mammalian HOPS complex on lysosomes
Source: J Cell Sci. 2015 May 1;128(9):1746–61. doi: 10.1242/jcs.162651 (PMC4432227; doi:10.1242/jcs.162651)
Supplement: Supplementary Material [file supp_128.9.1746_JCS162651.pdf]

Supplementary Figure Legends:

Figure S1: Vps41 localizes to lysosomes dependent upon GTP-bound Arl8b, but not Rab7. (A,B) HeLa cells co-transfected with HA-Vps41and Arl8b were co-stained with either LAMP2 or EEA1. Merge image shows colocalization of Vps41 and Arl8b with LAMP2 but not EEA1. **(C,D)** HeLa cells were co-transfected with HA-Vps41 and either Arl8b Q75L (C) or Arl8b T34N (D), and analyzed for their lysosomal localization by confocal microscopy. **(E,F)** Control or Rab7-silenced HeLa cells were transfected with GFP-RILP and immunostained with LAMP1, to visualize its localization by immunofluorescence **(G,H)** Control or Rab7 silenced HeLa cells were co-transfected with HA-Vps41 and Arl8b-tomato and analyzed for lysosomal localization by confocal microscopy. **(I)** Confocal micrograph depicting the colocalization of HA-Vps41 with GFP-RILP. **(**. Scale bar= 10 μM).

Figure S2: Subunit-subunit interaction analysis of the human HOPS complex and regulation of EGFR degradation by HOPS subunit hVps41. (A) Subunit-subunit interactions of all HOPS subunits was tested in a yeast-two-hybrid system by assaying for growth of co-transformants on non-selective medium, to confirm viability, and on selective medium containing 0.8mM 3-AT, to detect interactions. 3-AT was used to provide stringency, so as to prevent auto-activation of binding domain vector constructs. Yeast two-hybrid interaction of Vps11 was also tested in a separate experiment, where yeast were plated on selective medium (-His) without 3-AT so as to detect weaker interactions of Vps11 with other HOPS subunits. p53 (pVA3) and SV40 T-antigen (pTD1) are vectors provided by Clontech that contain fusion proteins that are able to interact with each other and were used as a positive control. **(B)** A model depicting the similar topological arrangement of *S. cerevisiae* HOPS and mammalian HOPS complex (based on the yeast-two-hybrid data). **(C)** HeLa cells were transiently co-transfected with Arl8b and multiple HOPS subunits in various combinations (label on x-axis), and number of co-localized endosomes of HOPS subunits with Arl8b^{+} were quantified. Values plotted are mean ± s.d. of three independent experiments (n=25 cells per experiment, * indicates $p<0.05$). **(D)** Control and Vps41-siRNA treated HeLa lysates were immunoblotted with anti-Vps41 and anti-actin antibodies. **(E)** An immunoblot of control or Vps41 shRNA-transduced HeLa cell lysates probed with Vps41 antisera or for tubulin (loading control). **(F-K)** Control (F-H) or Vps41-silenced (I-K) HeLa cells were serum starved and pulsed with Rhodamine-EGF (500ng/ml) for 7 minutes (F,I) and chased in complete medium for either 30 (G,J) or 60 minutes (H,K). EGFR levels remaining were

monitored over these time periods by staining with anti-EGFR antibody and lysosomes were visualized by anti-LAMP1 staining. Merge images are depicted. Scale bar= 10 μM.

Figure S3: Delay in trafficking of EGFR to the dextran-labeled compartment in Vps41-silenced cells. (A-D) Control (A,C) or Vps41-silenced (B,D) HeLa cells were either left untreated or incubated overnight with Alexa-488 labeled dextran (250μg/ml). Cells were further serum starved, pulsed with Rhodamine-EGF (500ng/ml) for 7 minutes and chased in complete medium for varying times. After fixation, the untreated set was immunostained with EEA1. Shown are representative images of EGF compartmentalization to EEA1-or dextran-labeled endosomes after 30 minutes of chase. Co-localized pixels are visualized in the inset. **(E)** Mander's and Pearson's coefficients were determined for EGF signal coincident with dextran signal. Dark gray and light gray bars represent control-siRNA and Vps41-siRNA treated cells, respectively. Values plotted correspond to mean ± s.d. of three independent experiments (n=50 cells per experiment, * and ** indicate $p<0.05$ and $p<0.01$, respectively). Scale bar= 10 μM.

Figure S4: Arl8b and SKIP-expression induces peripheral distribution of Vps41 and Vps39 endosomes, away from the perinculear-clustered RILP compartment. **(A-B)** Confocal micrographs depicting the localization of HOPS subunits Vps39 (A), and Vps41 (B), to SKIP, KLC2 and Arl8b-positive endosomes in transfected HeLa cells. **(C-F)** Control or Arl8b-silenced HeLa cells were transfected with Flag-SKIP and either HA-Vps39 or HA-Vps41, and visualized by immunofluorescence. **(G)** qRT-PCR analyses of SKIP levels in control and SKIP siRNA treated cells. **(H-K)** HeLa cells were transfected with GFP-RILP and HA-Vps39 (H), or GFP-RILP and HA-Vps41 (I) or, these constructs additionally with Flag-SKIP and Arl8b-tomato (J-K), and analyzed for their localization by confocal microscopy. Merge images represent localization of RILP and HOPS subunits. Scale bar= 10 μM.

Figure S1

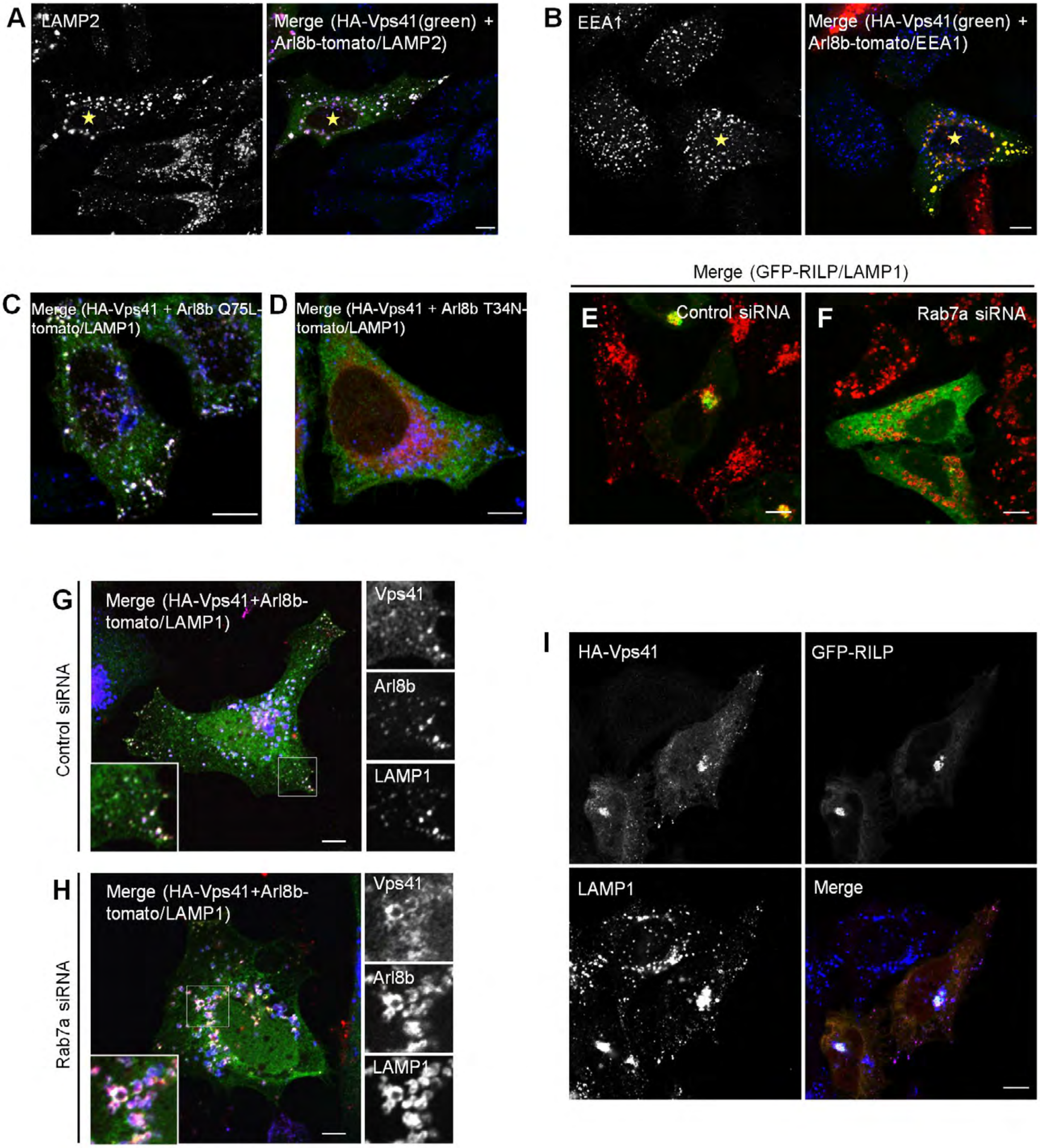
A
LAMP2
Merge (HA-Vps41(green) + Arl8b-tomato/LAMP2)
B
EEA1
Merge (HA-Vps41(green) + Arl8b-tomato/EEA1)
C
Merge (HA-Vps41 + Arl8b Q75L-tomato/LAMP1)
D
Merge (HA-Vps41 + Arl8b T34N-tomato/LAMP1)
Merge (GFP-RILP/LAMP1)
E
Control siRNA
F
Rab7a siRNA
G
Control siRNA
Merge (HA-Vps41+Arl8b-tomato/LAMP1)
Vps41
Arl8b
LAMP1
H
Rab7a siRNA
Merge (HA-Vps41+Arl8b-tomato/LAMP1)
Vps41
Arl8b
LAMP1
I
HA-Vps41
GFP-RILP
LAMP1
Merge

Figure S2

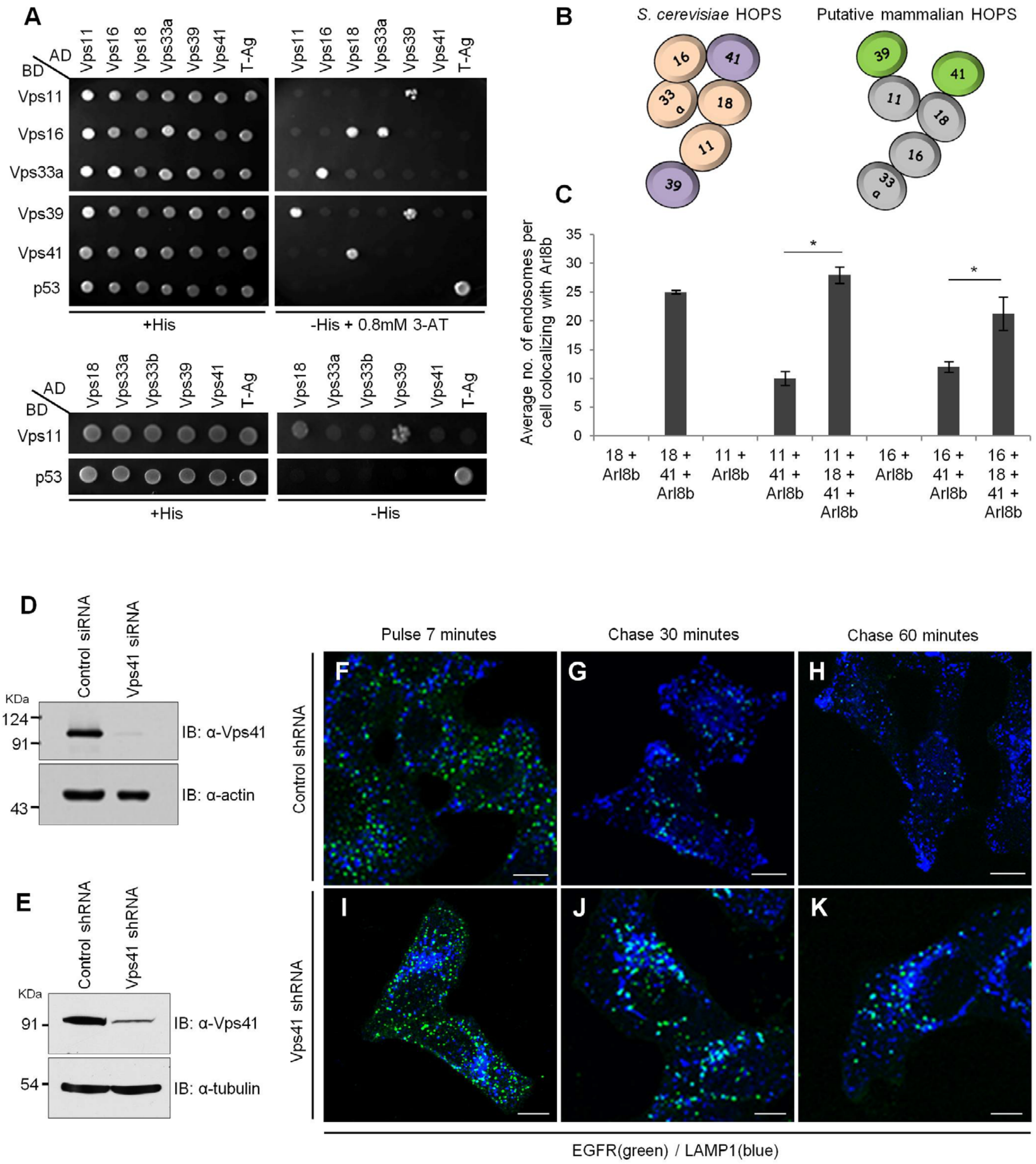

A
AD
BD
Vps11
Vps16
Vps18
Vps33a
Vps39
Vps41
T-Ag
p53
+His
-His + 0.8mM 3-AT
Vps33b
-His
B
S. cerevisiae HOPS
Putative mammalian HOPS
C
Average no. of endosomes per cell colocalizing with Arl8b
18 + Arl8b
18 + 41 + Arl8b
11 + Arl8b
11 + 41 + Arl8b
11 + 18 + 41 + Arl8b
16 + Arl8b
16 + 41 + Arl8b
16 + 18 + 41 + Arl8b
D
Control siRNA
Vps41 siRNA
KDa
IB: α-Vps41
IB: α-actin
E
Control shRNA
Vps41 shRNA
IB: α-tubulin
Pulse 7 minutes
Chase 30 minutes
Chase 60 minutes
F
G
H
I
J
K
EGFR(green) / LAMP1(blue)

Figure S3

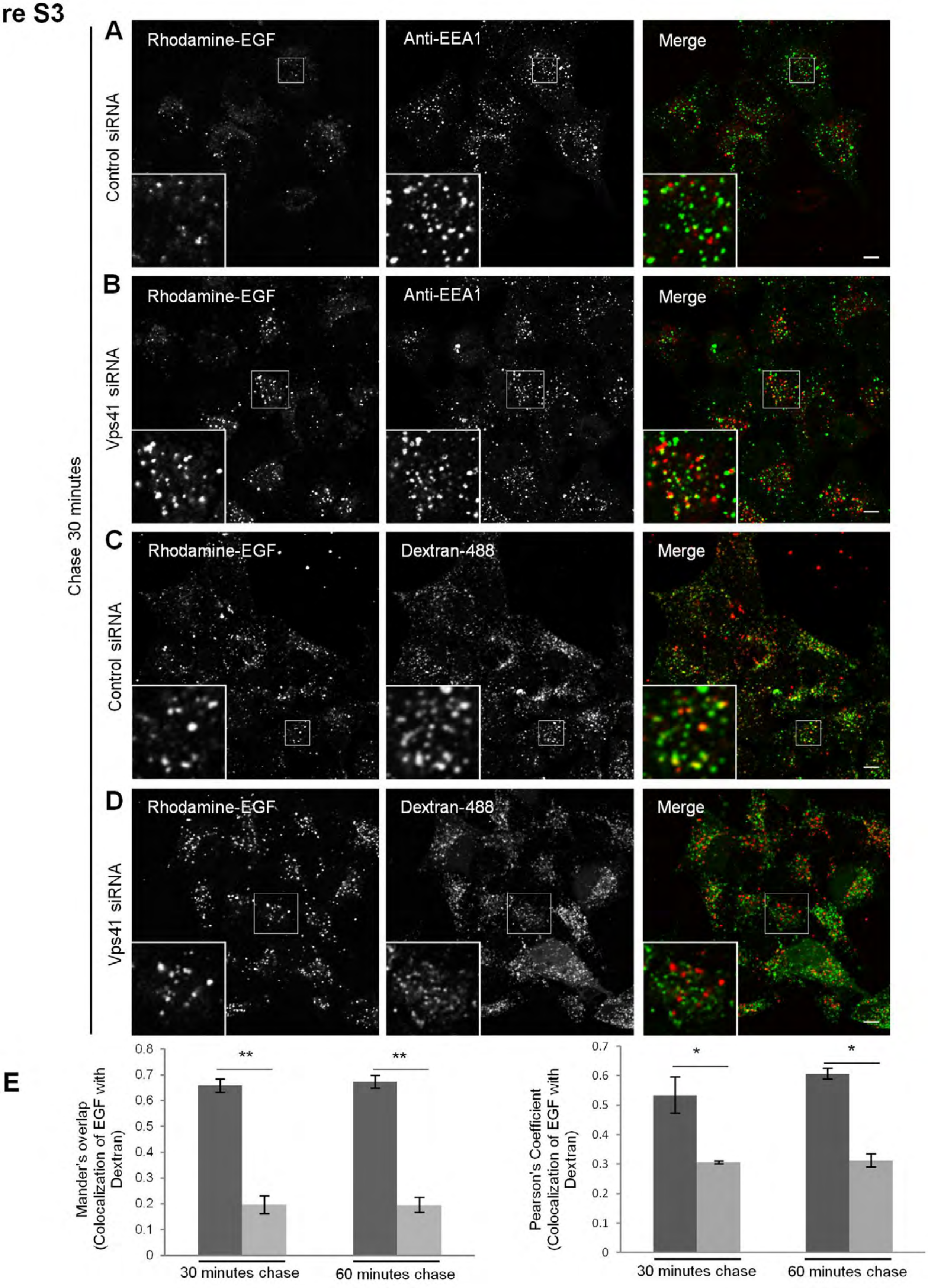
A
B
C
D
E
Rhodamine-EGF
Anti-EEA1
Dextran-488
Merge
Control siRNA
Vps41 siRNA
Chase 30 minutes
Mander's overlap (Colocalization of EGF with Dextran)
Pearson's Coefficient (Colocalization of EGF with Dextran)
30 minutes chase
60 minutes chase
**
*

Figure S4

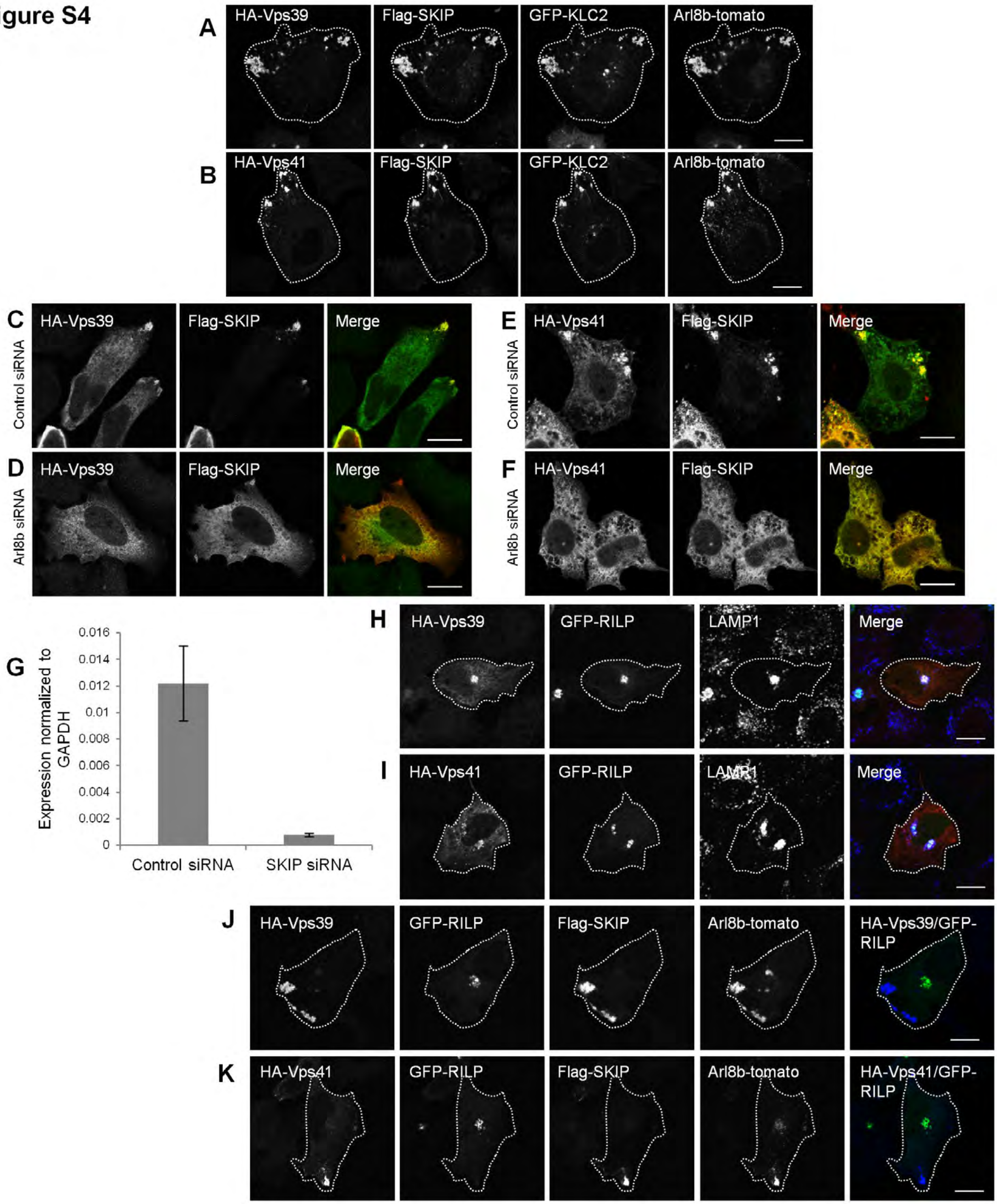
A
HA-Vps39
Flag-SKIP
GFP-KLC2
Arl8b-tomato
B
HA-Vps41
Flag-SKIP
GFP-KLC2
Arl8b-tomato
C
Control siRNA
HA-Vps39
Flag-SKIP
Merge
D
Arl8b siRNA
HA-Vps39
Flag-SKIP
Merge
E
Control siRNA
HA-Vps41
Flag-SKIP
Merge
F
Arl8b siRNA
HA-Vps41
Flag-SKIP
Merge
G
Expression normalized to GAPDH
0.016
0.014
0.012
0.01
0.008
0.006
0.004
0.002
0
Control siRNA
SKIP siRNA
H
HA-Vps39
GFP-RILP
LAMP1
Merge
I
HA-Vps41
GFP-RILP
LAMP1
Merge
J
HA-Vps39
GFP-RILP
Flag-SKIP
Arl8b-tomato
HA-Vps39/GFP-RILP
K
HA-Vps41
GFP-RILP
Flag-SKIP
Arl8b-tomato
HA-Vps41/GFP-RILP
